# The Adiponectin Receptor Homologs in *C. elegans* Promote Energy Utilization and Homeostasis

**DOI:** 10.1371/journal.pone.0021343

**Published:** 2011-06-21

**Authors:** Emma Svensson, Louise Olsen, Catarina Mörck, Christian Brackmann, Annika Enejder, Nils J. Faergeman, Marc Pilon

**Affiliations:** 1 Department of Cell and Molecular Biology, University of Gothenburg, Gothenburg, Sweden; 2 Department of Biochemistry and Molecular Biology, University of Southern Denmark, Odense, Denmark; 3 Department of Chemical and Biological Engineering, Chalmers University, Gothenburg, Sweden; Yale School of Medicine, United States of America

## Abstract

Adiponectin is an adipokine with insulin-sensitising actions in vertebrates. Its receptors, AdipoR1 and AdipoR2, are PAQR-type proteins with 7-transmembrane domains and topologies reversed that of GPCR's, i.e. their C-termini are extracellular. We identified three adiponectin receptor homologs in the nematode *C. elegans*, named *paqr-1*, *paqr-2* and *paqr-3*. These are differently expressed in the intestine (the main fat-storing tissue), hypodermis, muscles, neurons and secretory tissues, from which they could exert systemic effects. Analysis of mutants revealed that *paqr-1* and *-2* are novel metabolic regulators in *C. elegans* and that they act redundantly but independently from *paqr-3*. *paqr-2* is the most important of the three *paqr* genes: mutants grow poorly, fail to adapt to growth at low temperature, and have a very high fat content with an abnormal enrichment in long (C20) poly-unsaturated fatty acids when combined with the *paqr-1* mutation. *paqr-2* mutants are also synthetic lethal with mutations in *nhr-49*, *sbp-1* and *fat-6*, which are *C. elegans* homologs of nuclear hormone receptors, SREBP and FAT-6 (a Δ9 desaturase), respectively. Like *paqr-2*, *paqr-1* is also synthetic lethal with *sbp-1*. Mutations in *aak-2*, the *C. elegans* homolog of AMPK, or *nhr-80*, another nuclear hormone receptor gene, suppress the growth phenotype of *paqr-2* mutants, probably because they restore the balance between energy expenditure and storage. We conclude that *paqr-1* and *paqr-2* are receptors that regulate fatty acid metabolism and cold adaptation in *C. elegans*, that their main function is to promote energy utilization rather than storage, and that PAQR class proteins have regulated metabolism in metazoans for at least 700 million years.

## Introduction

Adiponectin is a hormone expressed specifically by adipocytes [1], [2], [3]. Its serum concentration correlates inversely with insulin resistance [4] and adipose mass [5], and low serum adiponectin level is a well-established risk factor for type 2 diabetes [6], [7], hepatic steatosis [8], [9] and myocardial infarction [10]. In mice, administration of adiponectin enhances insulin sensitivity and free fatty acid oxidation [11], [12], [13], [14], protects from atherosclerosis [15] and causes decreased body weight [12], [16]. Because of the wide-ranging potential for therapeutic benefits in human, adiponectin is now the subject of intense pre-clinical research.

Adiponectin exerts positive effects on peripheral metabolic tissues by promoting insulin sensitivity and energy expenditure. This is achieved via two homologous adiponectin receptors: AdipoR1 and AdipoR2 [17]. These are members of the poorly understood PAQR (progestin and adipoQ receptors) protein family characterized by seven transmembrane domains with a topology inverse that of G protein-coupled receptors (GPCRs): in PAQR proteins the N-terminus is intracellular [18]. Both receptors are expressed in many peripheral tissues [17], and in the hypothalamus where they regulate appetite [19]. Knockout mice have confirmed the insulin-sensitizing roles of these receptors: AdipoR1-deficient mice exhibit increased adiposity, insulin resistance and excess glucose production [20], [21], while AdipoR2 knockout mice show insulin resistance and reduced rates of glucose disposal and fatty acid oxidation [21]. These data suggest that activation of the adiponectin receptors regulates the balance between energy utilization and storage. Concordantly, binding of adiponectin to AdipoR1 leads to the activation of AMPK (AMP-activated protein kinase) in liver, which encourages catabolic processes, including the oxidation of fatty acids [13], [21]. AdipoR2, on the other hand, seems to act via PPARα (peroxisome proliferator-activated receptor alpha) to increase fatty acid oxidation, and increased expression of UCP-2 (uncoupling protein-2), which may protect cells from oxidative damage [21], [22]. In muscle, AdipoR1 induces PGC-1α (peroxisome proliferator-activated receptor gamma coactivator 1-α) and mitochondria biogenesis via Ca^2+^ and AMPK/SIRT1 signaling [23].

Recent work indicates that the AdipoR1/2 receptors have an associated ceramidase activity that may mediate much of their cellular effects [24], [25], [26]. However, events immediately downstream of adiponectin receptor activation are still poorly understood, partly owing to the difficulties in producing functionally active multimeric adiponectin, which curtails *in vitro* receptor activation experiments. No forward genetics model has yet been established to study these receptors, but their development would provide novel opportunities to decipher the downstream pathways. In the present study our aim was therefore to characterize the AdipoR1 and AdipoR2 homologs in *C. elegans*, an organism that has been instrumental in elucidating important aspects of the insulin-signaling pathway [27], [28], and that is increasingly used to understand lipid metabolism and storage. At least three receptor-based pathways regulate energy storage and utilization in *C. elegans* (reviewed in [29]): serotonin, insulin and TGF-β. Here we describe expression profiles, mutant phenotypes and genetic interaction studies for three *C. elegans* homologs of the human adiponectin receptors, with an emphasis on their roles as metabolic regulators.

## Results

### *paqr* genes: homology, structure and deletion alleles

**Figure 1:**
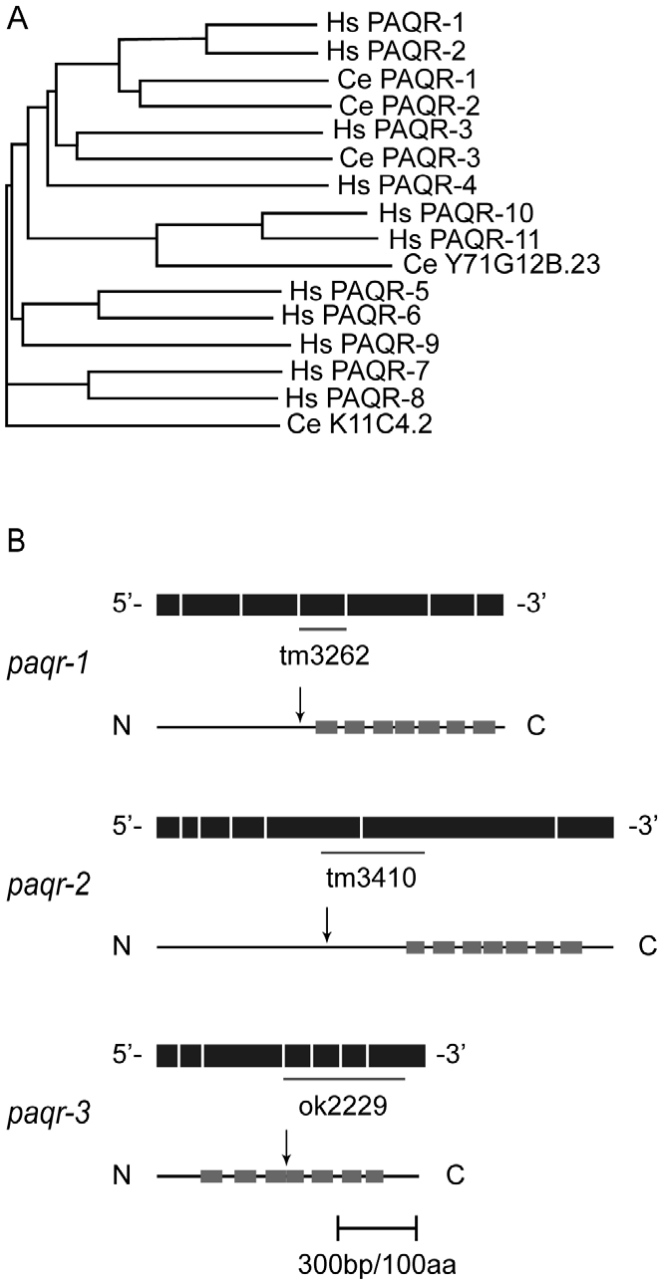
Relationship among *C. elegans* and human PAQR proteins. The dendogram was generated generated using Clustal W [30] with default settings to estimate the sequence homology between the eleven human PAQR proteins and the five *C. elegans* homologs. Hs PAQR1 and Hs PAQR2 correspond to ADIPOR1 and ADIPOR2, respectively.

While no obvious homolog of adiponectin exists in *C. elegans*, a search of its genome using the human receptor ADIPOR1 as query identified five genes with significant homology. Comparing these with all eleven human PAQR proteins using Clustal W [30] showed that the closest worm homologs of ADIPOR1 and ADIPOR2 are PAQR-1 (ORF C43G2.1) and PAQR-2 (ORF Y32H12A.5), while PAQR-3 (ORF Y67A10A.8) is more closely related to Hs PAQR-3 (Fig. 1; Supplementary Information S1 shows pair-wise comparisons of the amino acid sequences). The remaining two *C. elegans* proteins with homology to the human PAQRs are encoded by the ORFs Y71G12B.23 and K11C4.2 and are only distantly related to ADIPOR1. The *C. elegans paqr-1*, *-2* and *-3* genes each contain seven exons and encode a protein with seven transmembrane domains (Fig. 1 and Supplementary Information S1), and their gene structures have been confirmed by cDNAs (http://www.wormbase.org; version WS219).

### *paqr-1*, *-2* and *-3* have distinct expression patterns

**Figure 2:**
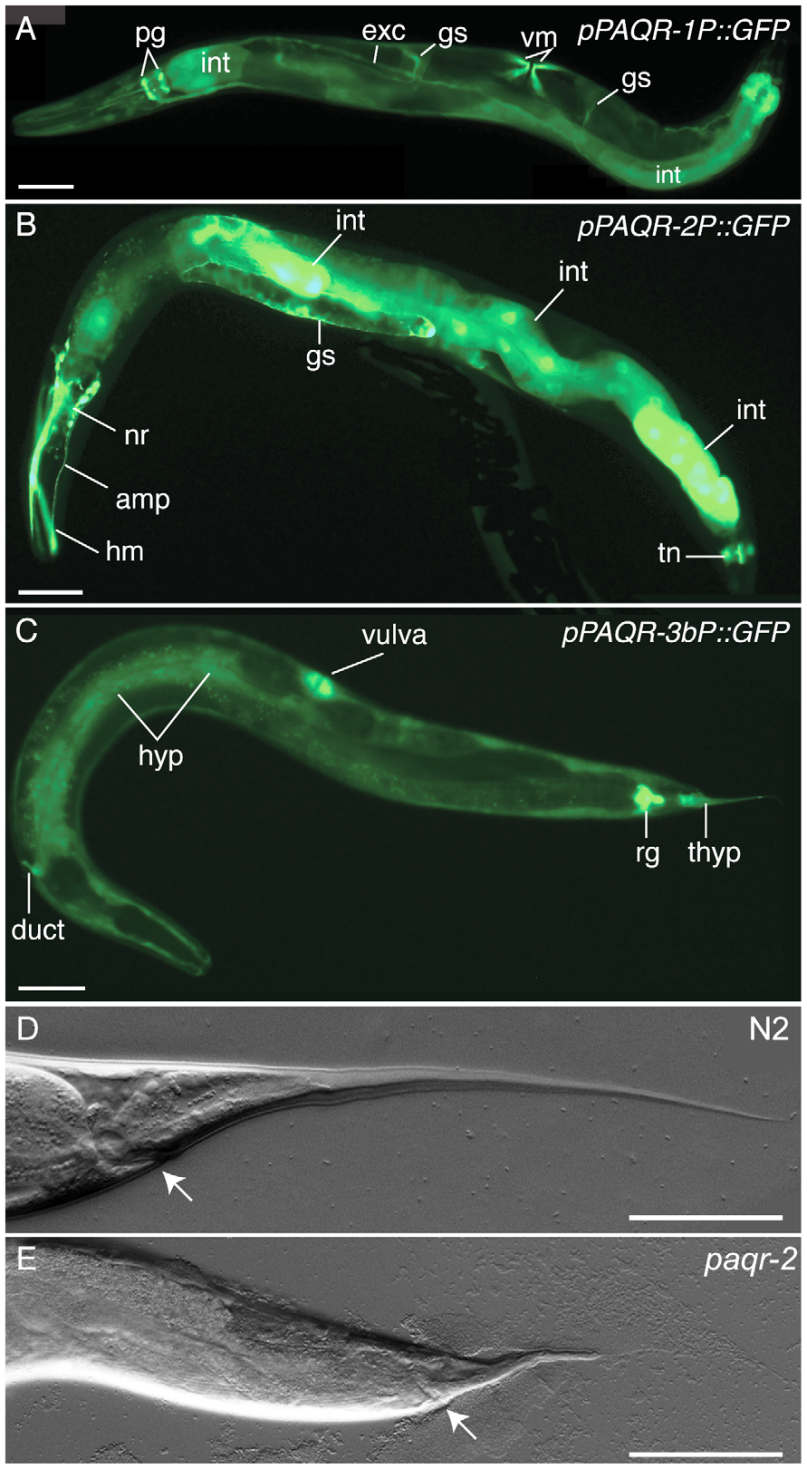
*paqr* gene expression and *paqr-2* tail tip phenotype. (A–C) Transgenic worms carrying the indicated transcriptional reporters are shown. Labels stand for the following: pg (pharyngeal gland), exc (excretory canal), gs (gonad sheath), vm (vulva muscle), int (intestine), hm (head muscle), amp (amphid neuron), nr (nerve ring), tn (tail neuron), hyp (hypodermis), rg (rectal gland) and thyp (tail hypodermis). *paqr-1P::GFP* and *paqr-3P::GFP* carry 1.5 kb of promoter while *paqr-3bP::GFP* carry 3 kb of promoter. Control hermaphrodites have smooth tails extending over 200 µm beyond the rectum (D) while *paqr-2* mutant hermaphrodites exhibits a withered tail tip phenotype (E) with a penetrance of 100%. Scales represent 50 µm.

Transcriptional reporters using ≥1.5 kb of upstream regulatory sequence from each *paqr* gene showed distinct expression profiles (Fig. 2A–C and Supplementary Information S1). *paqr-1* is consistently expressed in pharyngeal gland cells, excretory canal cell, vulva muscle, gonad sheath cell, intestine and occasionally in body muscles. *paqr-2* is expressed in head ganglion neurons, head muscle cells, the two pharyngeal M2 neurons, gonad sheath cell, seam cells, some ventral nerve cord and tail neurons, and occasionally in body muscles and intestine. *paqr-3* is weakly expressed only in the hypodermal cells. Because this gene is embedded within a dense cluster of short rRNA genes, we constructed a second construct harboring a longer (3.2 kb) stretch of the sequence upstream of the start codon to drive the *gfp* reporter. This showed *paqr-3* expression in hypodermal cells, duct cells, rectal gland, gonad sheath and vulva cells. Examination of embryos showed that expression of the reporters appeared gradually in the cells that would later remain *gfp*-positive in larvae and adults. With the caveat that our expression profiling is based on transcriptional reporters, it seems that the sites of expression are consistent with the *paqr* genes regulating the major metabolic organs (intestine, muscle and hypodermis), as well as possibly regulating systemic responses via the secretory systems and neurons.

### Phenotypic survey of the *paqr-1*, *-2* and *-3* single, double and triple mutants

**Figure 3:**
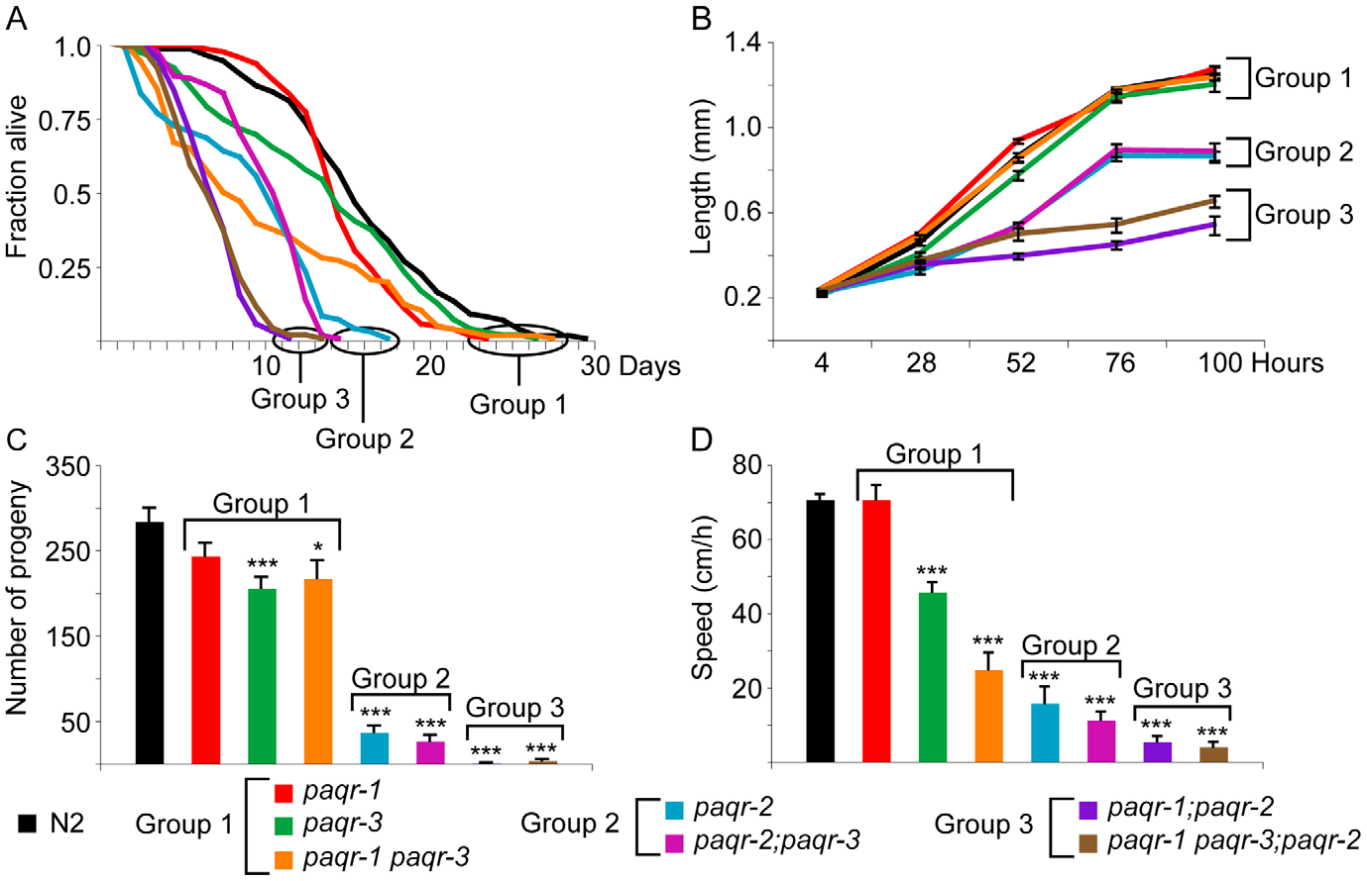
The various *paqr* genotypes fall into three phenotypic groups. Longevity (A), growth rate (B), brood size (C) and speed of locomotion (D) were determined in controls and all combinations of single and multiple *paqr-1*, *-2* and *-3* mutant genotypes. Note how the different genotypes can be grouped consistently across all phenotypic assay. *p<0.05, **p<0.01 and ***p<0.001; error bars show the sem.

**Figure 4:**
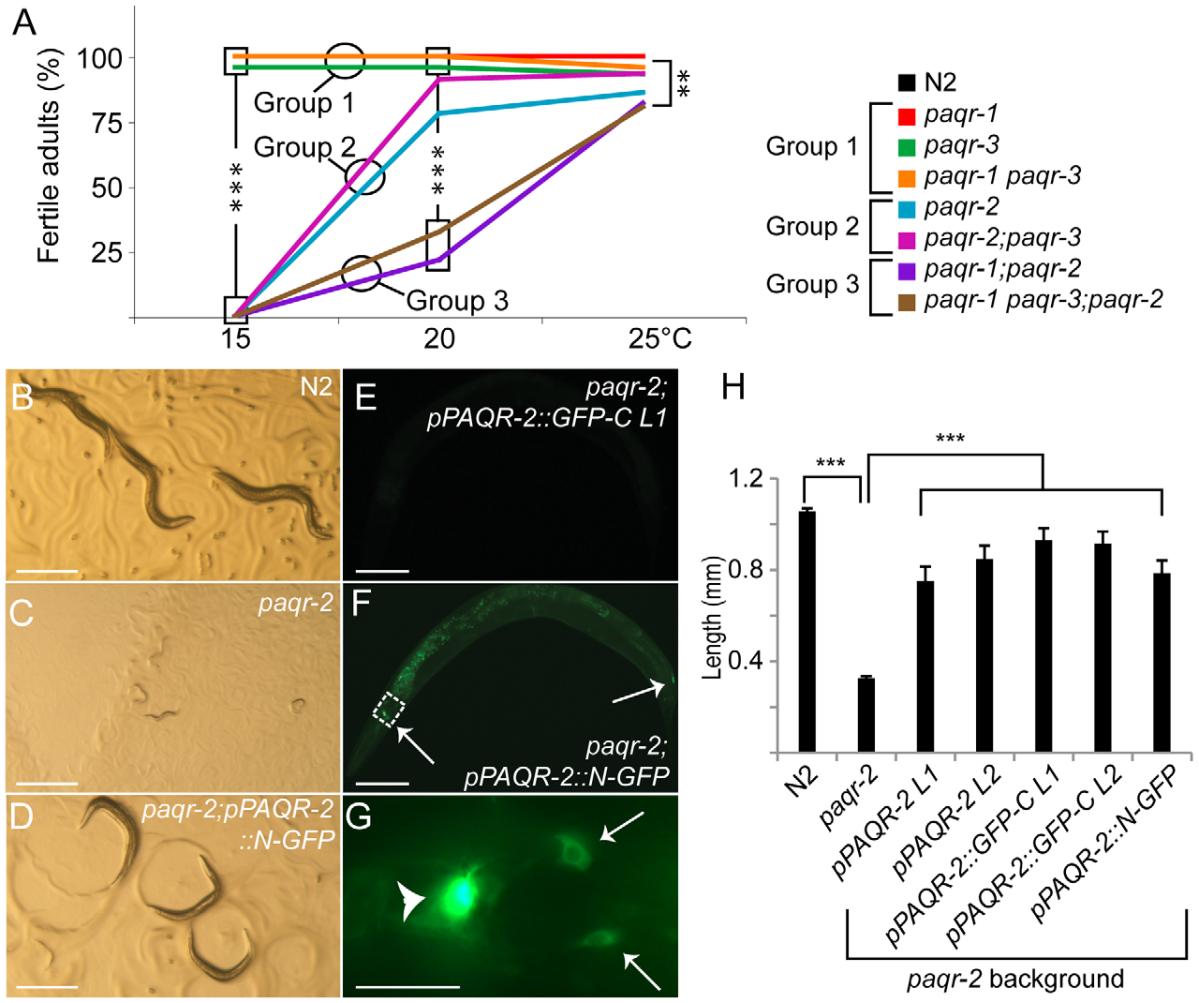
*paqr-2* is essential for growth at 15°C. (A) The fraction of individual L1 larvae that grew into fertile adults is shown for different cultivation temperatures. The grouped genotypes are the same as for Fig. 1. (B) and (C) Show wild type or *paqr-2* mutant worms grown for 120 hours at 15°C, while (D) shows the growth rescue of *paqr-2* worms carrying the *pPAQR-2::N-GFP* as a transgene. The *pPAQR-2::GFP-C* transgenics show no visible green fluorescence (E) while the *pPAQR-2::N-GFP* transgenics show expression of GFP on the plasma membranes of head and tail neurons as indicated by arrows in (F), with (G) being en enlarged view of the area boxed in (F) where the arrowhead indicates a bright neuron that is slightly out of focus. (H) Shows the average length of wild type worms, *paqr-2* mutant worms, and *paqr-2* mutants transgenic lines carrying the *pPAQR-2* transgene (2 lines, L1 and L2), the *pPAQR-2::GFP-C* transgene (2 lines, L1 and L2) and the *pPAQR-2::N-GFP* transgene (1 line). *p<0.05, ** p<0.01 and *** p<0.001 using the z-test in (A) and the unpaired Student's t-test in (H). Note that in (A) *paqr-2* is also significantly different from controls at 20°C and 25°C with p<0.01 and p<0.05, respectively. Scales indicate 500 µm (B–D), 100 µm (E–F) and 20 µm (G); error bars show the sem.

We obtained and characterized deletion mutant alleles for each *paqr* gene: *paqr-1(tm3262)*, *paqr-2(tm3410)* and *paqr-3(ok2229)*. One or more transmembrane domain is deleted in each mutant allele, and each deletion introduces premature stop codons and is therefore expected to severely disrupt protein function (Fig. 1). We initially compared wild-type and all combinations of single, double and triple *paqr* mutants. Only one morphological defect was observed among the mutants: *paqr-2* worms have a withered tail tip phenotype that is subtle during the early larval stages but becomes more pronounced in 100% of adults (Fig. 2D–E). Measurements of life span, growth rate, self-brood size and locomotion led to the interesting observation that the seven strains fall into three consistent phenotypic groups (Fig. 3). Group I includes the healthiest genotypes: *paqr-1*, *paqr-3* and *paqr-1 paqr-3*. Group II includes the genotypes *paqr-2* and *paqr-2*; *paqr-3*, and had reduced life span, growth rate, brood size and locomotion. Finally, Group III includes the genotypes *paqr-1*; *paqr-2* and *paqr-1 paqr-3;paqr-2*, which showed the severest phenotypes with very poor lifespan (average of about 7 days), growth rate (maximum size of about 600 µm after 4 days), brood size (less than 15 eggs produced per adult) and very poor mobility. The seven strains were also tested for the ability to grow from L1 to fertile adult at different temperatures, and again fell into the same three groups: Group I produced 100% fertile adults at all temperatures, Group II produced nearly 100% fertile adults at 20°C and 25°C, but 0% adults at 15°C, and Group III produced nearly 100% fertile adults at 25°C, fewer than 30% fertile adults at 20°C and 0% fertile adults at 15°C (Fig. 4).

It is evident that *paqr-2* is the most important of the *paqr* genes, and that its function is partially redundant with that of *paqr-1* but not of *paqr-3*. The most striking phenotype is the complete failure of any strain carrying *paqr-2* to grow at 15°C. This phenotype was rescued by reintroducing *paqr-2* as a transgene in the mutant, even when using GFP-tagged versions (Fig. 4). One version, *pPAQR-2-GFP-C*, carried the GFP sequence at the C-terminal end of the protein, while the other, *pPAQR-2-N-GFP*, carried the GFP sequence internally within the N-terminal domain some 90 amino acids away from the first transmembrane domain. Only *pPAQR-2-N-GFP* produced fluorescence, which was localized to the plasma membrane of several amphid neurons in the head (Fig. 4), as well as the DVC neuron of the dorsorectal ganglion, the PVT neuron of the preanal ganglion, one mid-body ventral nerve cord neuron and the gonad sheath (Supplementary Information S1).

In other tests, all three *paqr* single mutants performed normally for chemotattraction to isoamylalcohol and avoidance of a high molarity NaCl barrier. The three *paqr* mutants were also able to form dauer larvae when combined with the temperature sensitive *daf-2(e1370)* allele and grown at the non-permissive temperature of 25°C, and these dauer larvae were able to resume development when shifted back to the permissive temperature of 20°C. These phenotypes were not further investigated.

### *paqr* genes and lipid metabolism

**Figure 5:**
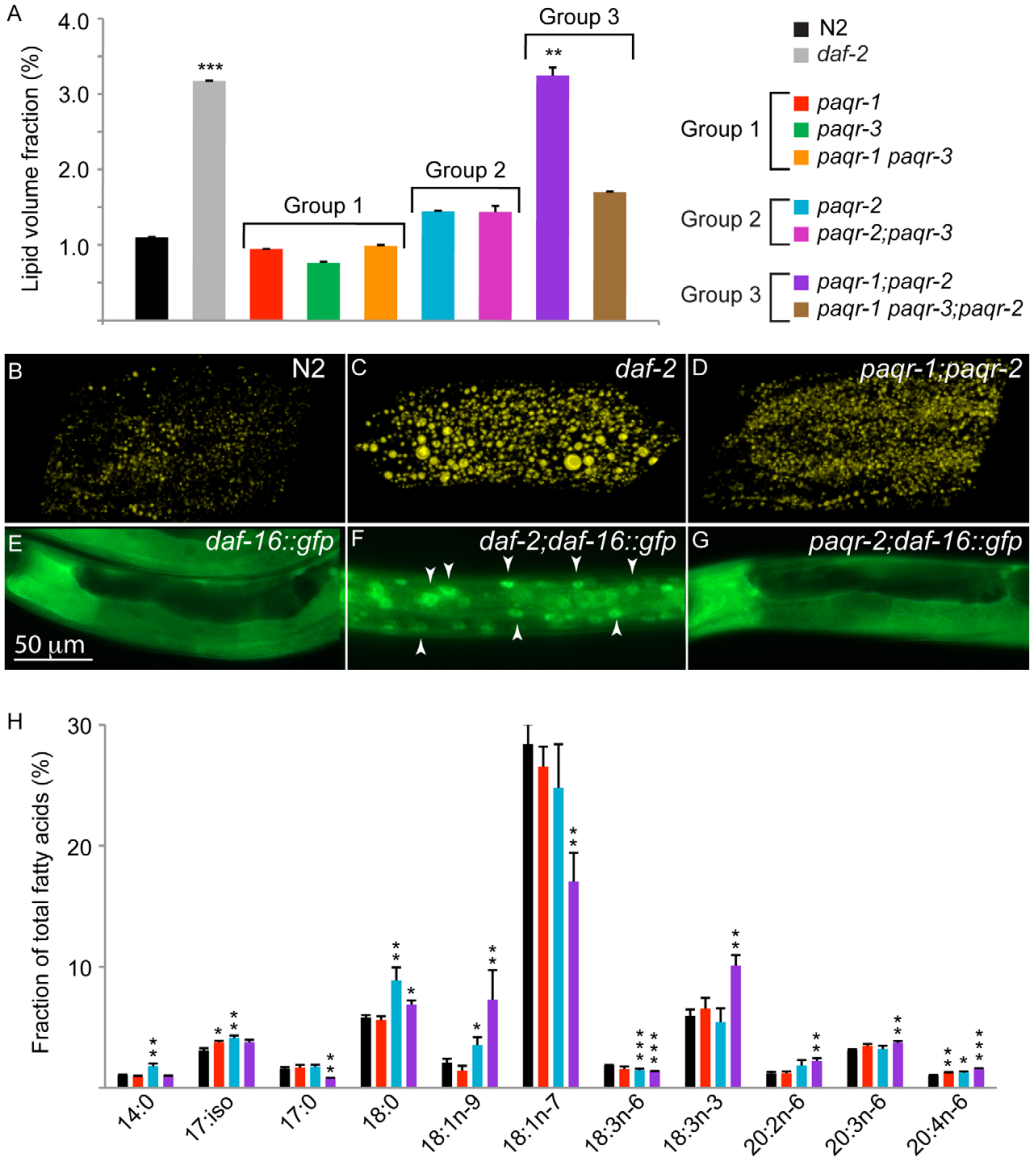
CARS microscopy and gas chromatography analysis of lipids. (A) Shows the fraction of the measured worm volumes occupied by lipid droplets (LVF). (B–D) show pictures of the worm with the LVF closest to the average for N2 (B), *daf-2* (C) and *paqr-1;paqr-2* (D). (E–G) Localization of DAF-16::GFP in N2, *daf-2* dauer larvae and *paqr-2* worms, respectively. Note the nuclear localization of DAF-16::GFP in the dauer larva (arrowheads in F) but not in control or *paqr-2* mutants. (H) Relative abundance of fatty acids that differed significantly between wild type and *paqr-1*, *paqr-2* or *paqr-1;paqr-2* mutants. * p<0.05, ** p<0.01, *** p<0.001 using Student's t-test in (A; test done on arc sin transformed data) and (H); error bars show the sem.

The adiponectin receptors regulate many aspects of lipid metabolism in mammals. Furthermore, poikilotherms such as *C. elegans* regulate the fatty acid composition of their membranes as they adapt to varying temperatures [31]. For these reasons, we explored the possibility that the *paqr* mutants, may exhibit defects in lipid metabolism which may account for the observed phenotypes, including failure of the *paqr-2* mutant to adapt to low temperature. We used Coherent Anti-Stokes Raman Scattering (CARS) microscopy to visualize lipid stores in the *paqr* mutants; CARS microscopy images fatty acid molecules directly without labeling by specific excitation of the C–H bonds [32], [33]. Three observations were clear from this analysis (Fig. 5): 1) none of the single mutants showed a significant difference in their lipid content compared to wild type; 2) the double mutant *paqr-1*; *paqr-2* had a highly elevated lipid content comparable to that of the *daf-2* mutant; and 3) the triple mutant *paqr-1 paqr-3*; *paqr-2* had a reduced lipid content compared to the double mutant *paqr-1;paqr-2*. These results suggest that *paqr-1* and *paqr-2* act redundantly to suppress the elevation of lipid stores, and that *paqr-3* may act in the opposite direction.

One way of quantifying droplet size is to determine their average area in each optical section [34]. We found that the average lipid droplet size was 0.95±0.03 µm^2^ for N2, 1.53±0.04 for *daf-2* and 1.00±0.12 for *paqr-1;paqr-2*. The *paqr-1;paqr-2* double mutant therefore has an increased number of small lipid droplets, while *daf-2* mutants have enlarged lipid droplets (Fig. 5B–D). The increased fat stores in the *daf-2* mutant depend on translocation of the forkhead transcription factor DAF-16 into the nucleus of somatic cells [27], [35]. Using a *daf-16::gfp* translational reporter [36], we saw no evidence of increased DAF-16 translocation to the nuclei in the *paqr-2* mutant (Fig. 5E–G), showing that this mutant induces increased fat stores in a pathway distinct from that engaged by *daf-2*.

Gas chromatography revealed several significant changes in the fatty acid composition of the *paqr* mutants (Fig. 5H; Table 1): Group I genotypes showed the least deviations from wild type, Group II showed a number of changes (8 of 19 fatty acids differed significantly from wild type), and Group III had significant and often dramatic changes in at least 10 of the 19 fatty acids monitored. The most significant changes in fatty acid composition concerned the 20-carbon polyunsaturated fatty acids: 4 out of 5 such fatty acids were highly elevated in the *paqr-1*; *paqr-2* and *paqr-1 paqr-3*; *paqr-2* mutants. Some of these lipids (e.g. arachidonic acid (C20:4n-6)) play important roles as components of cellular membranes or in cellular signaling [37]. The proportion of arachidonic acid and dihomo-γ linoleic acid (C20:3n-6) has also been shown to decrease when *C. elegans* are grown at low temperatures [31].

**Table 1:** Fatty acid composition of total lipid fraction for wild type and *paqr* mutants at 20°C.

		Group 1			Group 2		Group 3	
Fatty acid	N2	*paqr-1*	*paqr-3*	*paqr-1 paqr-3*	*paqr-2*	*paqr-2; paqr-3*	*paqr-1; paqr-2*	*paqr-1 paqr-3; paqr-2*
C14:0	1.0±0.1	0.9±0.1	1.0±0.2	0.9±0.1	1.8±0.2**	2.3±0.5**	1.0±0.0	1.0±0.0
C15:iso	3.5±0.1	3.2±0.2	3.4±0.2	3.4±0.1	3.8±0.5	3.9±0.5	3.2±0.0	3.4±0.1
C16:0	4.5±0.2	3.8±0.2*	4.1±0.4	4.1±0.5	4.7±0.4	6.2±0.6**	4.0±0.2	3.1±0.1***
C16:1n-7	3.0±0.4	2.1±0.3	2.4±0.2	2.4±0.1	2.3±0.6	3.0±0.9	1.7±0.2	1.7±0.2
C17:iso	3.1±0.2	3.8±0.1*	3.6±0.3	3.8±0.2*	4.1±0.2**	4.4±0.5**	3.8±0.2	4.6±0.1**
C17:0	1.6±0.1	1.7±0.2	1.6±0.1	1.6±0.1	1.7±0.2	2.6±0.7	0.7±0.1**	0.7±0.1**
C17Δ	16.6±0.8	16.0±0.5	16.4±0.8	14.5±0.4	12.4±1.3*	10.2±1.7**	15.7±0.1	12.9±0.5*
C18:0	5.8±0.2	5.6±0.3	5.6±0.5	5.8±0.7	8.9±1.1**	11.8±1.8***	6.9±0.3*	6.2±0.1
C18:1n-9	2.1±0.3	1.4±0.4	2.6±0.7	2.1±0.5	3.5±0.7*	3.0±0.4	7.3±2.4**	2.6±0.4
C18:1n-7	28.4±1.6	26.5±1.6	26.0±2.2	26.2±1.7	24.8±3.6	24.5±4.0	17.0±2.4**	21.0±0.3*
C18:2n-6	3.3±0.2	3.2±0.4	3.8±0.5	4.0±0.3	4.3±0.4*	3.5±0.6	3.3±0.4	3.7±0.5
C19Δ	2.7±0.3	3.4±0.3	3.5±0.5	2.7±0.2	3.2±0.6	2.8±0.7	2.8±0.2	2.0±0.3
C18:3n-6	1.9±0.0	1.6±0.2	1.7±0.1	1.9±0.1	1.5±0.1***	2.3±0.6	1.3±0.1***	1.5±0.0***
C18:3n-3	5.9±0.5	6.6±0.9	6.0±0.6	5.6±0.4	5.4±1.1	3.7±0.8*	10.1±0.9**	8.9±0.9*
C20:2n-6	1.2±0.1	1.2±0.2	1.4±0.3	1.7±0.3	1.9±0.4	1.1±0.5	2.2±0.2**	2.7±0.3***
C20:3n-6	3.1±0.1	3.5±0.2	3.3±0.1	3.7±0.1**	3.2±0.3	3.0±0.5	3.7±0.1**	5.0±0.2***
C20:4n-6	1.0±0.0	1.3±0.0**	1.2±0.1*	1.6±0.1***	1.2±0.1*	1.3±0.1**	1.6±0.1***	2.6±0.1***
C20:4n-3	3.2±0.2	3.9±0.5	3.4±0.2	3.6±0.1	2.9±0.2	2.6±0.3	3.4±0.1	2.8±0.1
C20:5n-3	8.0±0.4	10.3±0.7*	8.9±0.6	10.5±0.5**	8.2±0.6	7.7±0.6	10.3±0.6*	13.6±0.6***

Values are weight percentages, mean ± SEM. n  =  10 for wild type and 5–6 for *paqr* mutants.

*p<0.05;

**p<0.01;

***p<0.001 with Students t-test.

### Dietary rescue of *paqr-2* phenotypes

**Figure 6:**
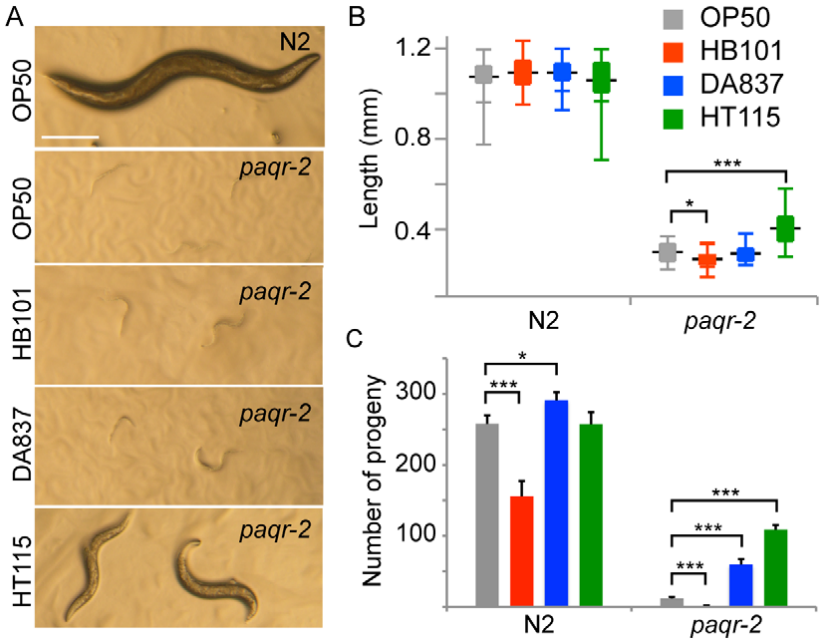
Partial rescue of *paqr-2* mutant growth at 15°C and brood size at 20°C using different diets. (A) Shows wild type or *paqr-2* worms grown at 15°C for 6 (N2) or 11 (*paqr-2*) days using various types of bacteria as food sources. Note how HT115 visibly rescues the growth of the *paqr-2* mutant. Scalebar is 250 µm and applies to all images. (B) Length at day 6 for wild type or *paqr-2* mutants grown at 15°C on the indicated food sources; boxes span from the 25^th^ to the 75^th^ percentile, outer brackets indicate the range of length and the horizontal black line indicates the mean length for each genotype (n≥19). (C) Shows the average brood sizes obtained for wild type or *paqr-2* worms grown at 20°C on the indicated food sources, and the error bars show the sem. *p<0.05, **p<0.01, ***p<0.001.

Some of the phenotypes observed in *paqr-2* mutants probably reflect the defect in fatty acid composition, which leads to the hypothesis that modulating fatty acids may rescue these phenotypes. A simple way to modulate the lipid composition in *C. elegans* is to use different types of bacteria as food sources [38]. We compared four *E. coli* strains for their ability to rescue the *paqr-2* phenotypes: OP50 (the standard *C. elegans* diet), HT115 (a strain with higher carbohydrate content), HB101 (high content of carbohydrates, increased monounsaturated fatty acids and decreased cyclopropane fatty acids) and DA837 (larger physical size than OP50, but with a similar carbohydrate, fatty acid and protein content) [38]. As described earlier, the growth and reproduction of *paqr-2* mutants is inhibited at 15°C when grown on the standard OP50 diet. This phenotype was partially rescued by the HT115 diet (Fig. 6A–B). *paqr-2* mutants grown at 20°C on DA837 or HT115 bacteria were also more successful at completing their life cycle and laying eggs compared to when fed the standard OP50 diet (Fig. 6C). Interestingly, *paqr-2* mutants seem unaware of the benefits of the DA837 or HT115 diets since they do not prefer them over OP50 (Supplementary Information S1).

### Genetic interactions identify metabolic pathways regulated by *paqr-2*

Genetic interaction studies were used to better define the nature of the metabolic defects in the *paqr-2* mutant. Three types of genes were tested: regulators of fatty acid metabolism, their effectors, and genes implicated in ceramide signaling (Supplementary Information S1). The regulation of fatty acid metabolism in *C. elegans* relies on several signaling molecules such as *sbp-1* (the sterol regulatory element-binding protein/SREBP homolog; [39], [40], [41]), *nhr-49* and *nhr-80* (nuclear hormone receptors; [42], [43]), and *aak-2* (AMPK homolog; [29]). Among the effectors are the fatty acid Δ9 desaturases *fat-5*, *fat-6*, and *fat-7* [29], [43], [44], and the acyl-CoA synthethase *acs-2* which is involved in fatty acid β-oxidation [44]. Finally, recent reports suggest that PAQR proteins may signal through ceramides [25], [26], [45], and this led us to generate double mutants involving the acid ceramidase F27E5.1 as well as the ceramide synthases *hyl-1*, *hyl-2* and *lagr-1*.

**Figure 7:**
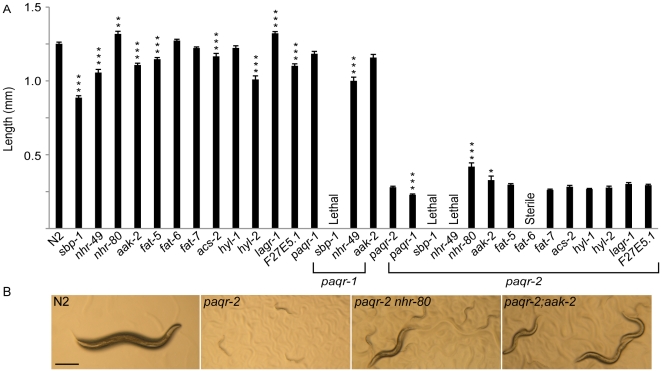
Genetic interaction studies involving *paqr-1* or *paqr-2*. (A) Shows the average length of the indicated genotypes six days after plating as synchronized L1 larvae and cultivated at 15°C. (B) Representative images of the indicated genotypes. Note the rescue of growth in the *paqr-2 nhr-80* and *paqr-2;aak-2* double mutants compared to the *paqr-2* single mutant. >20% of rescued double mutants grew to adulthood at 15°C compared to 0% for *paqr-2* single mutants (n>200). Scalebar is 250 µm and applies to all images. *p<0.05, **p<0.01, ***p<0.001; error bars show the sem. Significance refers to the comparison with the relevant genetic background, i.e. single mutants are compared to N2, double mutants involving *paqr-1* or *paqr-2* are compared to the *paqr-1* or *paqr-2* single mutant, respectively.

The double mutants involving *paqr-2* and either *fat-5*, *fat-7*, *acs-2*, *F27E5.1*, *hyl-1*, *hyl-2* or *lagr-1* are viable and show no enhancement of the *paqr-2* growth phenotype (Fig. 7). *paqr-2* is therefore not acting redundantly in an essential process with any one of these genes. Strikingly, we were unable to establish a viable strain doubly homozygous for mutations in *paqr-2* and any of the following genes: *nhr-49*, *sbp-1* or *fat-6*. Worms homozygous for *paqr-2* and heterozygous for *nhr-49* produced a low frequency of sickly progeny that typically arrested in the early larval stages, although some rare decrepit individuals managed to grow and lay a few progeny; these sickly worms proved to be double homozygous for *paqr-2* and *nhr-49* when scored by PCR. Similarly, double homozygotes for *paqr-2 sbp-1* typically failed to grow to the adult stage and were always sterile, while *paqr-2;fat-6* typically grew to adulthood but were always sterile. *paqr-2* therefore acts redundantly in essential processes with *nhr-49*, *sbp-1* and *fat-6*. Double mutants between *paqr-1* and *nhr-49* or *aak-2* were viable, but *paqr-1;sbp-1* double mutants typically died as larvae and the few individuals that grew to adulthood were always sterile. Conversely, mutations in the genes *aak-2* or *nhr-80* rescued the growth phenotype of the *paqr-2* mutant, suggesting that these have effects opposite that of *paqr-2* (Fig. 7).

## Discussion

Based on the sequence homologies and their effects on fatty acid metabolism it seems clear that the *C. elegans paqr-1* and *paqr-2* are the orthologs of the mammalian AdipoR1 and AdipoR2 genes, and that their main function is to promote energy utilization rather than storage. Based on transcriptional reporters, *paqr-1* and *paqr-2* are expressed in muscles and intestine, which are the main sites of fatty acid oxidation and storage, respectively. *paqr-2* also stands out for its expression in several neurons. Thus, the *paqr-1* and *paqr-2* genes are well positioned to regulate fat storage and oxidation, and even to influence behavior systemically, as they do in mammals. Our results unambiguously establish *paqr-1* and *paqr-2* as a new receptor-based pathway that fills an explanatory gap in the regulation of fatty acid synthesis and oxidation in *C. elegans* by genetically interacting with *nhr-49*, *nhr-80* and *sbp-1*, for which receptor-based regulatory pathways had not yet been identified. [29].

### paqr-1 and paqr-2 as metabolic regulators

Like the *daf-2* mutant, that have defects in insulin signaling, *paqr-1;paqr-2* double mutants have an increased fat content. However, the nature of this increase differs between the mutants: lipid droplets are larger but not more numerous in the *daf-2* mutant compared to wild-type, while they are more numerous but not larger in the *paqr-1;paqr-2* double mutant. This suggests that the *paqr-1* and *-2* genes are regulators of lipid droplet biogenesis or turnover, and that the *paqr* and *daf-2* genes lead to increased fat via separate pathways. This is consistent with the fact that we saw no nuclear localization of a *daf-16::gfp* reporter in *paqr-2* mutants, while it is well known that *daf-2* mediates increased fat storage by activating *daf-16* [27]. Other pathways that could lead to an increase in fat storage are *daf-7/TGF-β* via the *daf-1/4* receptor, serotonin via the *mod-1* receptor, and a pathway involving the mediator *mdt-15* acting together with the transcription factors *sbp-1* and *nhr-49* [29].

**Figure 8:**
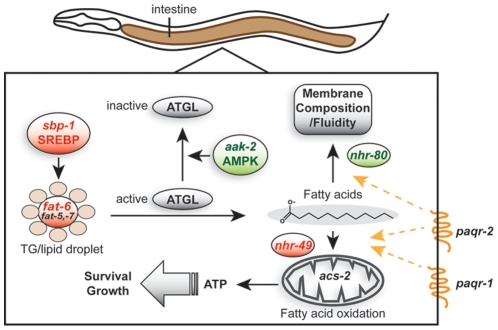
Speculative model of genetic interactions involving the *paqr-1* and *paqr-2* genes. This model could explain the observed genetic interactions in the following way: 1) *paqr-1* or *paqr-2* are synthetic lethal with *sbp-1* (or *fat-6*) due to reduced fatty acid synthesis/TAG storage (reduced substrate supply) and reduced fatty acid oxidation, leading to ATP shortage; 2) *paqr-2*;*nhr-49* is lethal because two redundant pathways regulating fatty acid oxidation are impaired; 3) *paqr-2;aak-2* are healthier than the *paqr-2* single mutant because the *aak-2* mutation causes increased lipolysis, thus providing more fatty acids for energy production; and 4) *paqr-2 nhr-80* double mutants have decreased fatty acid desaturation (since *nhr-80* is a positive regulator of *fat-5* and *fat-7)*, which may result in improved membrane fluidity at 15°C. Mutations in genes highlighted in red were synthetic lethal with *paqr-2* while those in green produced genetic rescues.

In Fig. 8 we present a genetic interaction model as the basis for discussion. While the interpretation of our results should be done cautiously in view of the pleiotropic phenotypes, the simplest explanation for the observed genetic interactions is that *paqr-1* and *paqr-2* function by promoting fatty acid oxidation, thus shifting the energy balance from storage to expenditure. If that is the case, then the *paqr-2 sbp-1* and *paqr-2;fat-6* double mutants are lethal because of reduced fatty acid synthesis/TAG storage (reduced substrate supply) combined with reduced fatty acid oxidation, leading to ATP shortage. That is because the *C. elegans* gene *sbp-1* is a positive regulator of the fatty acid Δ9 desaturases *fat-5*, *-6* and *-7*, and that its activity encourages the storage of fat [40]. Our results also show that *nhr-49* and *nhr-80* have some opposite effects since the double mutant *paqr-2;nhr-49* is not viable even at 20°C while the double mutant *paqr-2 nhr-80* is rescued and able to grow at 15°C. The *nhr-49* and *nhr-80* genes encode nuclear hormone receptors distantly related to PPARα and that differ in their functions: while both regulate *fat-5* and *-7* to promote storage of fatty acids, *nhr-49* also regulates *fat-6*, and acts as a positive regulator of *acs-2* and *ech-1* to promote fatty acid oxidation by mitochondria [43], [46]. Since *paqr-2* and *nhr-49* may have similar roles in regulating fatty acid oxidation, it is plausible that this process is more severely affected in the double mutant, leading to drastic reduction in ATP synthesis, thus explaining the lethality of the double mutant. Conversely, mutations in *nhr-80* may rescue growth of *paqr-2* mutants at 15°C because of a continued expression of *fat-6* combined with reduced expression of *fat-5* and *fat-7* which should result in a decreased abundance of polyunsaturated fatty acids that may benefit membrane composition/fluidity at low temperatures. The effects of poor energy utilization in the *paqr-2* mutant were also genetically rescued by a mutation in *aak-2*, a *C. elegans* AMPK homolog that has been shown to inhibit the adipose triglyceride lipase *atgl-1* [47], [48]. When *aak-2* is mutated in the *paqr-2* background, *atgl-1* activity would increase, leading to increased lipolysis, fatty acid oxidation and ATP synthesis, hence survival.

### paqr-2 and cold adaptation

That the *paqr-2* mutant is unable to grow at 15°C is intriguing. Poikilotherms must adjust the lipid compositions of their membranes as the temperature of their environment changes. Assuming that the mutant's inability to grow on the *E. coli* strain OP50 at 15°C is not due to changes in the *E. coli* bacteria themselves [49], the likeliest explanation is that the *paqr-2* mutant is unable to make a successful cold-adaptation. Several of the lipids that we identified as being present in abnormal quantities in the *paqr-2* mutant are important for the modulation of membrane fluidity in response to temperature changes in *C. elegans* [31]. The HT115 diet partially rescued the growth inhibitory effects of cultivating *paqr-2* mutants at the low temperature of 15°C, and rescued also the small brood size at 20°C. HT115 may be rich in lipids or carbohydrates that facilitate adjustments in membrane composition [31] as well as other changes [50]. The fact that *paqr-2 nhr-80* double mutants could grow at 15°C partly supports this hypothesis. Could adiponectin receptors play an evolutionarily conserved role in adaptation to temperature changes, and could such a role be conserved even in homotherms such as mammals? This is indeed a possibility as judged by the acute changes in circulating adiponectin levels in mice and men when exposed to cold stress [51], [52].

### Structure-Function of PAQR-2

Our results with GFP-tagging of the PAQR-2 protein are consistent with the topology of PAQR proteins, with the N-terminus being cytoplasmic and the C-terminus being extracellular [18]. GFP-tagging the PAQR-2 protein internally within the cytoplasmic N-terminal domain, but outside the region conserved with human AdipoR2, produced a fluorescent protein enriched on the plasma membrane and that was functional since able to rescue the *paqr-2* mutant. GFP-tagging PAQR-2 at its C-terminus also produced a functional protein that could rescue the *paqr-2* mutant, but this tagged version is not fluorescent presumably because of misfolding as it becomes the extracellular tail of the protein. These results, which are the first to functionally test GFP-tagged version of adiponectin receptors, show that no essential domains are present at the C-terminal end of the protein, nor in the intracellular region just outside the conserved stretch of about 90 amino acids that lies next to the first transmembrane domain.

### Developmental function of paqr-2

The *paqr-2* mutant exhibits a withered tail tip phenotype. This defect appears different from that observed in other mutants with a related withered tail phenotype because it affects only the tip of the tail, and the animals show no associated “clear” phenotype [53], [54]. In any case, the phenotype suggests some developmental or hypodermal cell maintenance function for *paqr-2*. The mouse AdipoR1 gene may also have a developmental function since a knockout mutant exhibited an enlarged brain phenotype and also had testis developmental defects that caused sterility [20]. These phenotypes were however not reported for a separately generated AdipoR2-deficient mouse, and the basis for the discrepancy is not known [21].

### Screen paqr-2 suppressors

One of the merits of model organisms such as *C. elegans* is the ease with which genetic screens can be performed. Given the inability of *paqr-2* mutants to grow at 15°C it should be relatively straightforward to isolate suppressor mutations that allow growth at that temperature, leading to the identification of downstream targets of *paqr-2*. The present study therefore lays the foundation for unbiased genetics approaches to unravel the mechanisms of action of PAQR proteins such as the adiponectin receptor homologs.

## Materials and Methods

### *C. elegans* cultivation, strains and transgenes

Maintenance of worms were performed as described elsewhere [55]. The wild type reference strain was the *C. elegans* Bristol variety strain, N2. The mutants listed in Supplementary Information S1 were obtained from the *C. elegans* Genetics Center (CGC; MN, USA), except for *sbp-1(ep79)* which was provided by J. Watts (Wash. State Univ., Pullman, USA). The transgene *zIs356 [pGP30 (daf-16::gfp) pRF4 (rol-6)]* was also obtained from CGC, from strain TJ356. The bacterial strains OP50, DA837 and HB101 were obtained from CGC, while the strain HT115 was from the Ahringer RNAi library [56]. We also obtained the *paqr-1(tm3262)* and *paqr-2(tm3410)* deletion mutants from the National Bioresource Project for the Experimental Animal “Nematode *C. elegans*”. The *paqr-3(ok2229)* mutant allele was obtained from the *C. elegans* Knock-Out Consortium. The *paqr* mutants were each outcrossed ten times with N2 worms prior to this study. The molecular nature of the three *paqr* mutants is described in Wormbase [57]. The detection of the *paqr* mutant alleles by PCR, and the construction of reporter plasmids and rescuing constructs are described in Supplementary Information S1.

### Generation of transgenic animals

Germline transformation and use of the dominant *rol-6 (su1006)* marker for identifying transgenic worms were as previously described [58].

### Fluorescent and Differential Interference Contrast (DIC) microscopy

Animals were placed on 2% agarose pads, on glass slides in a drop of 10 mM levamisole as anaesthetic and overlaid with a cover slip. Animals were observed with a Zeiss Axiophot microscope using a GFP filter or DIC optics. Images were taken using the Axiovision 4.5 program (Zeiss) and further processed using Photoshop (Adobe).

### CARS microscopy and gas chromatography

The CARS microscopy and gas chromatography analysis of total lipids on animals grown at 20°C was performed as previously described (see Supplementary Information S1) [32], [33].

### Life span assay

Synchronous L4s were plated in groups of 5 worms onto NGM plates. The worms were transferred every second day during the fertile period and once a week thereafter. All worms were monitored every day and scored as dead when failing to respond upon several touches on the head with the worm-pick.

### Brood size assay

Synchronous L1s were plated onto NGM plates seeded with OP50, for assaying the *paqr* strains, and OP50, HB101, HT115 or DA837 for the diet rescue assay. When grown to L4 stage 10–23 worms were singled out onto new plates of the corresponding diet. The worms were transferred daily during the fertile period and live progeny were counted 3 days after removal of the hermaphrodite.

### Growth assay

Synchronized L1s were plated onto NGM plates seeded with the assay diet. For the growth rate assay 10–18 worms were mounted and photographed after 4, 28, 52, 76 and 100 h. For all other assays 19–26 worms were mounted and photographed after 5 (rescue of *paqr-2* with various transgenes) or 6 days. Worm length (excluding the thin tail tip) was measured using ImageJ [59].

### Locomotion assay

Synchronized L1s were plated, picked when L4s and used for the assay as young adults the next day. Three worms were started at different places on a seeded 10 cm diameter NGM plate. Their tracks were followed for 30 min and drawn on the back of the plate. The length of the tracks were measured to scale in ImageJ [59], 5–6 worms were assayed/strain.

### Dietary choice assay

This assay was performed essentially as described elsewhere [60]. Briefly, NGM plates lacking peptone were seeded using a computer made template as a guide to obtain a 6 mm distance between spots of bacteria. Synchronized L1s were plated in 1.5 µl M9. Plates were incubated at the assay temperature and the worms on 3 plates/treatment were killed by chloroform vapour at each time point (1, 4, 8, and 24 h).

## Supporting Information

Supplementary Information S1 Supplementary Materials and Methods, figures, and tables.
(PDF)
