# Supplementary material for: The Adiponectin Receptor Homologs in C. elegans Promote Energy Utilization and Homeostasis
Source: PLoS One. 2011 Jun 21;6(6):e21343. doi: 10.1371/journal.pone.0021343 (PMC3119701; doi:10.1371/journal.pone.0021343)
Supplement: Supplementary Information S1 — Supplementary Materials and Methods, figures, and tables. (PDF) [file pone.0021343.s001.pdf]

SUPPLEMENTARY INFORMATION

SI Materials and Methods

PCR detection of the *paqr* mutant alleles

The *paqr-1(tm3262)* allele carries a 538 bp deletion + 2 bp insertion such that its sequence now reads: ...taacttcaaaatggtctctaTT(538_bp_deletion)ttgattttttattatttta..., where the uppercase "TT" indicates the two inserted nucleotides. The following primers were used to distinguish the wild type and mutant loci: 5'-cgtcgggaatagaacgatca-3' and 5'-atagaacggcaccagcgaag-3', which give a 1 495 bp band in wild type and a 959 bp band in mutant.

The *paqr-2(tm3410)* allele carries a 772 bp deletion + 6 bp insertion such that its sequence now reads: ...atggggacgagtcagaaaGAAAAA(722_bp_deletion)gtccccga-cgttgtggat..., where the uppercase "GAAAAA" indicates six inserted nucleotides. The following primers were used to distinguish the wild type and mutant loci: 5'-tgggagatcgcgtgataatg-3' and 5'-ggagatgtcccatcaacgtc-3' which give a 1 777 bp band in wild type and a 1011 bp band in mutant.

The *paqr-3(ok2229)* allele carries a 1 522 bp deletion + 1 bp insertion such that its sequence now reads: ...gactgaagaagaagagatttT(1522_bp_deletion)actaaaatgtttaca-atttt..., where the uppercase "T" indicates the inserted nucleotide. The following primers were used to distinguish the wild type and mutant loci: 5'-ctagaattccccgacttccc-3' and 5'-aattcacgagccgatttttg-3' which give a 3 333 bp band in wild type and a 1800 bp band in mutant.

Construction of transcriptional reporters and rescuing plasmids

The *pPAQR-1P::GFP* transcriptional reporter was constructed by first amplifying 1.5 kb of sequence upstream of the start codon using the primers 5'-ggatcctcgattgacctcatctgga-3' (BamHI site underlined) and 5'-ctgcagagaacgtagagtctctggga-3' (PstI site underlined) and lysed N2 worms as source of template. The resulting PCR product was cloned into the *pCR-Blunt II-TOPO* vector (InVitrogen), then subcloned as a PstI-BamHI fragment into the corresponding sites of *pPD95.77* (1) to produce a GFP reporter driven by the *paqr-1* regulatory region, and flanked at its 3' end by the *unc-54* 3' UTR.

The *pPAQR-2P::GFP* and *pPAQR-3P::GFP* transcriptional reporters were similarly constructed but using instead the following primer pairs: 5'-ggatcccgattccacgtcatcttcctcca-3' (BamHI site underlined) together with 5'-ctgcagtggaatggcttgaggatctcgca-3' (PstI site underlined) and 5'-ggatccttgatacctctcatgattatggca-3' (BamHI site underlined) together with 5'-ctgcagctcatccatctcgttgatggt-3' (PstI site underlined). *pPAQR-3bP::GFP*, which carries 3.2 kb of sequence upstream of the start codon of *paqr-3*, was similarly constructed by using 5'-ctgcagactggctattgtcagaacaga-3' (PstI site underlined) as the left primer.

The *pPAQR-2* construct carries the genomic version of the *paqr-2* gene, and was generated using the primers 5'-gacaggaagtgttctcattcgt-3' and 5'-actgaggagcaacaagtgaaca-3' to amplify the 3' end of the *paqr-2* gene (~2kb) and about 1.1kb of 3' UTR with a mixture of N2 worms as template. The resulting ~3.1kb PCR product was subcloned into *pCR-Blunt II-TOPO*, digested with SalI and NotI and

ligated into the corresponding sites of TOPO_fragment A (an intermediate plasmid during the construction of *pPAQR-2::GFP-C* described below).

The *pPAQR-2::GFP-C* rescuing plasmid, which carries the GFP sequence at the C-terminal end of the PAQR-2 protein coding region, was constructed by using the primers 5'-gtctagatggaatggcttgaggatctcgca-3' (XbaI site underlined) and 5'-gtctagaaaaccaacatccgccggtgtcca-3' (XbaI site underlined) to amplify the *paqr-2* gene and 1.5kb of upstream sequence with a mixture of N2 worms as template. The resulting ~5.2kb PCR product was subcloned into *pCR-Blunt II-TOPO* (making TOPO_fragmentA), digested with XbaI and ligated to the Fire vector *pPD95.75* (1).

The *pPAQR-2::N-GFP* construct, which carries the GFP sequence internally after amino acid 195 within the cytoplasmic N-terminal domain of the PAQR-2 protein coding region, was generated using the primers 5'-agtcgacaaatgagtaaaggagaagaact-3' (SalI site underlined) and 5'-agtcgactgtatagttcatccatgcca-3' (SalI site underlined) to amplify the GFP sequence using *pPD95.75* as template. The resulting 866bp PCR product was subcloned into *pCR-Blunt II-TOPO*, digested with SalI and ligated into the corresponding site of *paqr-2_pBSKS* (itself made by transferring the *paqr-2* promoter, gene and UTR from *pPAQR-2* as a SpeI-ApaI fragment into the corresponding sites of *pBSKS*).

CARS microscopy

Specifically, the setup employed for the Coherent Anti-Stokes Raman Scattering (CARS) microscopy consists of an inverted microscope (Nikon, Eclipse TE2000-E) and a near-infrared laser system (a Nd:Vanadate laser, HighQ Laser Production GmbH), seeding a Levante Emerald Optical Parametric Oscillator, (APE GmbH) generating two synchronized pico-second pulsed beams at 1064 nm and 817 nm, respectively. The beams are overlapped and guided into the microscope via a mirror scanning unit and sequentially scanned over the sample. A motorized focusing stage mounted with a high numerical aperture objective (Nikon Plan Flour, 40x, oil, N.A. 1.3) allows 3-dimensional sectioning. The two excitation beams form a beating field in the focal volume with a frequency that matches a vibrational resonance of the acyl CH_2 groups at 2845 cm^{-1}, characteristic for lipids. At sample positions with high concentrations of lipids, a strong CARS-signal is generated and collected in the forward direction by a condenser lens (N.A. 0.8) mounted on the microscope. Single CARS photons are finally registered by a photomultipier tube (PMC-100-1, Hamamatsu) equipped with band-pass filters and subsequently counted by a time correlated single photon counting device (Becker and Hickl GmbH, SPC-830). This allows the distribution of lipids in the sample to be mapped into a 3-dimensional volume image.

Nematodes (2-day adults) to be analyzed were placed in a droplet of Levamisole (10 mM) on a 0.17 mm thick microscopy cover glass covered with a thin 2% agarose pad containing 0.2% 1-phenoxy-2-propanol. A second cover glass was gently applied and the distribution of lipid droplets was mapped in the first intestinal ring, just posterior to the pharynx of the larva . For each specimen, a z-stack of 40-50 CARS microscopy images was collected, covering 100×100 µm in the xy-plane (256×256 pixels) and 40-50 µm in depth. The lipid droplets in each stack were isolated by means of local (dynamic) thresholding as described by Hagmar *et al.* (2), and quantitatively evaluated with respect to Lipid Volume Fraction (LVF), and droplet size (μm^2). A

square-shaped region-of-interest in the central position of the nematode was selected, containing both lipids of the hypodermal and the intestinal region. The same evaluation area was then applied to all images in the stack containing lipid droplets. The LVF was computed by summing up the relative area covered by lipid droplets in the region-of-interest for each plane.

Gas chromatography. L4 larvae were harvested, washed and resuspended in freshly prepared 2.5% H_2SO_4 in methanol (1 ml) supplemented with BHT (10 µg/ml), and incubated for 5 hours at 80°C. Subsequently, the fatty acid methyl esters were extracted by the addition of hexane (0.5 ml) and H_2O (1.5 ml). The organic phase was transferred to fresh sample vials and dried under a stream of N_2. Each sample was redissolved in hexane and FAMEs were analysed by gas chromatography on a Chrompack CP 9002 equipped with a DB-WAX (127-7012, Agilent Technologies, USA) column. The FAMEs were identified by comparison to standards purchased from Larodan (Malmö, Sweden).

References

1. Fire A, Harrison SW, & Dixon DA (1990) A modular set of *lacZ* fusion vectors for studying gene expression in *Caenorhabditis elegans*. *Gene* 93:189-198.
2. Hagmar J, Brackmann C, Gustavsson T, & Enejder A (2008) Image analysis in nonlinear microscopy. *J Opt Soc Am A Opt Image Sci Vis* 25(9):2195-2206.
3. Thompson JD, Higgins DG, & Gibson TJ (1994) CLUSTAL W: improving the sensitivity of progressive multiple sequence alignment through sequence weighting, position-specific gap penalties and weight matrix choice. *Nucleic Acids Res* 22(22):4673-4680.
4. Liang B, Ferguson K, Kadyk L, & Watts JL (2010) The role of nuclear receptor NHR-64 in fat storage regulation in Caenorhabditis elegans. *PLoS One* 5(3):e9869.
5. Apfeld J, O'Connor G, McDonagh T, DiStefano PS, & Curtis R (2004) The AMP-activated protein kinase AAK-2 links energy levels and insulin-like signals to lifespan in C. elegans. *Genes Dev* 18(24):3004-3009.
6. Brock TJ, Browse J, & Watts JL (2006) Genetic regulation of unsaturated fatty acid composition in C. elegans. *PLoS Genet* 2(7):e108.
7. Deng X*, et al.* (2008) Ceramide biogenesis is required for radiation-induced apoptosis in the germ line of C. elegans. *Science* 322(5898):110-115.

LEGENDS TO SUPPLEMENTARY FIGURES S1-S6

Fig. S1. Structures of the *C. elegans* PAQRs and comparison with their human homologs. (A) Structures of the *C. elegans paqr-1, -2* and *-3* genes and proteins. Exon boundaries are indicated by white lines, gray boxes indicate transmembrane domains and arrows indicate the position of predicted stop codons in the mutants. (B) Annotated amino acid sequence of PAQR-1 and comparison with the human ADIPOR1 protein. *paqr-1* encodes a protein of 434 amino acids with homology to ADIPOR1 and ADIPOR2. The predicted dimerization motif is shown in bold within the fifth transmembrane domain. The cytoplasmic N-terminal end of the PAQR-1 protein is longer than that of ADIPOR1 (200 aa versus 134 aa). Note how the homology between the *C. elegans* PAQR-1 protein and ADIPOR1 extends throughout the transmembrane domains and over the 64 amino acids of the cytoplasmic N-terminus that are closest to the membrane. The amino acids in red are deleted in the *tm3262* allele. This allele has a deletion that begins and ends within the introns flanking exon 4, and would also introduce a stop codon at aa 177 in the aberrant open reading frame even if alternatively spliced from exon 3 to exon 5. (C) Annotated amino acid sequence of PAQR-2 and comparison with the human ADIPOR2 protein. *paqr-2* encodes a protein of 581 amino acids with homology to ADIPOR1 and ADIPOR2. The predicted transmembrane domains are underlined. The predicted dimerization motif is shown in bold within the fifth transmembrane domain. The cytoplasmic N-terminal end of the *paqr-2* protein is longer than that in ADIPOR2 (313 aa versus 148 aa). Note how the homology between the *C. elegans* PAQR-2 protein and ADIPOR2 extends throughout the transmembrane domains and over the 90 amino acids of the cytoplasmic N-terminus that are closest to the membrane. The amino acids in red are deleted in the *tm3410* allele. This allele has a deletion that begins in exon 5 and ends in exon 6; this would create a novel reading frame after the deletion which would introduce a stop codon at aa 212 of the aberrant frame. In the construct *pPAQR-2::N-GFP*, the GFP sequence was inserted between the two arginine indicated in blue, i.e. within the cytoplasmic N-terminal domain, but outside the sequence conserved between PAQR-2 and ADIPOR2. (E) Annotated amino acid sequence of PAQR-3 and comparison with the human PAQR3 protein. *paqr-3* encodes a protein of 581 amino acids with high homology to human PAQR3, and weaker homology to ADIPOR1 and ADIPOR2. Note how the homology between the *C. elegans paqr-3* encoded protein and PAQR3 extends throughout the protein. The amino acids in red are deleted in the *ok2229* allele; this allele has a deletion that begins in an intron and ends in an exon, deleting exons 4-6 and more than half of exon 7, which is the last one in the transcript. The deletion introduces a stop codon at aa 163 of the expected aberrant open reading frame. The predicted transmembrane domains are underlined.

Fig. S2. Expression pattern of *paqr-1*. The photographs show GFP expression in animals transgenic for a *paqr-1* promoter reporter in which 1.5 kb of sequence upstream of the start ATG, and the first few *paqr-1* codons are fused in frame with the GFP reporter gene. (A) Shows an entire worm, while (B-E) shows enlarged views of specific GFP-positive structures, with a DIC view provided in the right column. Abbreviations are as follows: bm (body muscles), exc (excretory cell), gs (gonad

sheath cell), int (intestine), pg (pharyngeal gland), sp (spermatheca), vm (vulva muscles). All scales represent 25 μm.

Fig. S3. Expression pattern of *paqr-2*. The photographs show GFP expression in animals transgenic for a *paqr-2* promoter reporter in which 1.5 kb of sequence upstream of the start ATG, and the first few *paqr-2* codons are fused in frame with the GFP reporter gene. (A) Shows an entire worm, while (B-G) shows enlarged views of specific GFP-positive structures, with a DIC view provided in the right column. Abbreviations are as follows: amp (amphid neuron), bm (body muscle), dtc (distal tip cell), gs (gonad sheath), hg (head ganglion), hm (head muscle), int (intestine), M2 (M2 neuron), nr (nerve ring), seam (seam cells), sp (spermatheca), tn (tail neurons), vnc (ventral nerve cord). All scales represent 25 μm.

Fig. S4. Expression pattern of *paqr-3*. The photographs show GFP expression in animals transgenic for a *paqr-3* promoter reporter in which 3 kb of sequence upstream of the start ATG, and the first few *paqr-3* codons are fused in frame with the GFP reporter gene. (A) Shows an entire worm, while (B-F) shows enlarged views of specific GFP-positive structures, with a DIC view provided in the right column. Abbreviations are as follows: duct (duct cell; the pore cell may also be GFP-positive), gc (gonad cytoplasm), hyp (hypodermal cells), rg (rectal gland), thyp (tail hypodermal cell), vulva (vulva cells, likely uv3). All scales represent 25 μm.

Fig. S5. Expression pattern of a PAQR-2::GFP translational fusion reporter. All animals shown are transgenic for the plasmid *pPAQR-2-N-GFP.* (A-C) Shows the head region where four GFP-positive amphid neurons are indicated by an arrow or arrowheads in B, only one of which also stains with DiI (arrow in B and C). (D-E) Shows the tail region with the position of the anus indicated by the arrow. The dorsorectal ganglion neuron DVC is GFP-positive in E, and distinct from the DiI labeled phasmid PHA and PHB neurons of the tail (F). Occasionally, the two other dorsorectal ganglion neurons (DVA and DVB) are also GFP-positive. (G-H) Shows GFP expression in an unidentified lateral neuron as well as the gonad sheath (gs). (I-J) Shows GFP expression in the PVT neuron of the preanal ganglion, and again in DVC.

Fig. S6. Food preference assay for wild type and *paqr-2* mutants. The fraction of worms found on either food type over time is shown for each test, with the grey area indicating the fraction of worms on the standard OP50 food. The high fraction of *paqr-2* worms off the food at the early time points likely reflects the poor locomotion of these mutant worms. *$p<0.05$, **$p<0.01$, ***$p<0.001$ indicate significant difference between N2 and *paqr-2* for the same time point and food combination.

FIG S1

(A)

```
PAQR-1    MNPDEVNRALGHYLNDADSGELVVEDSTTVQVKNPEARKTGDKIEVFYSRKTTVVSPTNS -60
AdipoR1   --------------MSSHKGSVVAQGNGAPASNREADTVELAELGPLLEEKGKRVIANPP -46
                         .:..*.:*.:.. :   :.        ::  : ..* . * .. .

PAQR-1    DDEDADFCSDSELLPQQEGHRSRATSFAGRIRAGSDDEAMPKHTILRYRRKKGGQWREIN -120
AdipoR1   KAEEEQTCP----VPQEEEEEVRVLTLP-------------------------------- -70
          . *: : *.    :**:* .. *. ::.

PAQR-1    LQGTPDKRKDDEDELEVDVKEDRSEQTGIVTKTYEARWKVLKYEHLPEWLQDNEFLRHGH -180
AdipoR1   ----------------LQAHHAMEKMEEFVYKVWEGRWRVIPYDVLPDWLKDNDYLLHGH -114
                          ::.:.  .:   :* *.:*.**:*: *: **:**:**::* ***

PAQR-1    RPPLPSFSECFKSIWSLHTETGNIWTHLIGCVAFFFLACWFLTRP--DNHIQFQEKVVFS -238
AdipoR1   RPPMPSFRACFKSIFRIHTETGNIWTHLLGFVLFLFLGILTMLRPNMYFMAPLQEKVVFG -174
          ***:***  *****: :***********:* * *:**.   : **       :******.

PAQR-1    FFFAGAVLCLGLSFAFHTLSCHSVNVVKIFCKLDYMGISLLIIGSFIPWIYYGFYCRREP -298
AdipoR1   MFFLGAVLCLSFSWLFHTVYCHSEKVSRTFSKLDYSGIALLIMGSFVPWLYYSFYCSPQP -234
          :** ******.:*: ***: *** :* : *.**** **:***:***:**:**.***  :*

PAQR-1    KITYIAMVSVLGIGAIVVSLWDKFSESRFRPIRAAVFVGMGCSGVIPTIHYIITDGVHSL -358
AdipoR1   RLIYLSIVCVLGISAIIVAQWDRFATPKHRQTRAGVFLGLGLSGVVPTMHFTIAEGFVKA -294
          :: *:::*.****.**:*: **:*: .:.*  **.**:*:* ***:**:*: *::*. .

PAQR-1    FADNSFHWLLLMAFLYLLGAGLYATRTPERFFPGKCDIWFQSHQLFHTCVVIAAFVHYYG -418
AdipoR1   TTVGQMGWFFLMAVMYITGAGLYAARIPERFFPGKFDIWFQSHQIFHVLVVAAAFVHFYG -354
           : ..: *::***.:*: ******:* ******** ********:**. ** *****:**

PAQR-1    ISEMAFARLNEQCPVR-----                                        -434
AdipoR1   VSNLQEFRYGLEGGCTDDTLL                                        -375
          :*::   * . :
```

FIG. S1 (cont'd)

(B)

```
PAQR-2     MEEDDVESATPAESQKLLQKSVRNSFDERDMVMVLDNDELIMENRNSSNLQSPLRSVTDV -60
ADIPOR2    ------------------------------------------------------------ -


PAQR-2     LREAENIITDGPLQLADTQENQNVIVDQVDVQFPNSQPSDGQRFEIIVRHSKVVEQPASQ -120
ADIPOR2    ----MNEPTENRLGCSRTPEP--------DIRLRKGHQLDGTRRGDNDSHQGDLEPILEA -48
                *  *:. *  : * *         *::: :.:  ** *      *.  :*   .

PAQR-2     QRDESMYVTRQNRLPTLLEGIEIDNGDVPSTSKAAALPFKKGHRRAWSMPNAKGEKMTVA -180
ADIPOR2    SVLSSHHKK--------------------------------------------------- -57
           .  .* : .

PAQR-2     VIHDNHEEGGNQHRRRIVKYKLHPQRRGHEDGKHLRFVTAGHDAEIHLDHSDDDDELEVE -240
ADIPOR2    ------------------------------------------SSEEHEYSDEAPQEDEGF -75
                                                     .:* *   .:  :* *

PAQR-2     INEEEVIIPSETGEGPRAVIKRFWEARWKATNFETLPEWLQDNEYLRTGHRPPLPSFSSC -300
ADIPOR2    MGMSPLLQAHHAMEKMEEFVCKVWEGRWRVIPHDVLPDWLKDNDFLLHGHRPPMPSFRAC -135
           :. . :: . .: *  . .: :.**.**:.  .:.**:**:**::*  *****:*** :*

PAQR-2     FKSIFALHTETGNIWTHMYGCVAFFGIGIWFLTRP--HDQMQWVEKLIYSAFFLGAVICL -358
ADIPOR2    FKSIFRIHTETGNIWTHLLGCVFFLCLGIFYMFRPNISFVAPLQEKVVFGLFFLGAILCL -195
           ***** :**********: *** *: :**::: **         **:::. *****::**

PAQR-2     GMSFLFHTVACHSIEMAKLFSKLDYTGISLLIVGSFVPWIYFAFYCRPQPMIIYNGMIII -418
ADIPOR2    SFSWLFHTVYCHSEGVSRLFSKLDYSGIALLIMGSFVPWLYYSFYCNPQPCFIYLIVICV -255
           .:*:***** ***  :::*******:**:***:******:*::***.*** :**  :* :

PAQR-2     LGIAAMVVSLWDKFAEPKFRPVRAAVFIAMGLSAVVPAFHLLVVDGVHYMVEESQVHLMI -478
ADIPOR2    LGIAAIIVSQWDMFATPQYRGVRAGVFLGLGLSGIIPTLHYVISEGFLKAATIGQIGWLM -315
           *****::** ** ** *::* ***.**:.:***.::*::* :: :*.   .  .*:  ::

PAQR-2     MMGAMYIGGATLYATRVPERCFPGKCDIWFQSHQLFHTFVVLAAFVHYHAISQMGMHKLM -538
ADIPOR2    LMASLYITGAALYAARIPERFFPGKCDIWFHSHQLFHIFVVAGAFVHFHGVSNLQEFRFM -375
           :*.::** **:***:*:*** *********:****** *** .****:*.:*::  .::*

PAQR-2     KGSCPDQLKTRYGNELNPSFVGTLLGLDEPLNVRDWTPADVGL                  -581
ADIPOR2    IGGGCSEEDAL--------------------------------                  -386
            *.  .: .:
```

FIG. S1 (cont'd)

(C)

```
PAQR-3        MRKRCLKCNLKDVCHNHERY--------RLVSKHKLHKTLWQNDYVINHYRPPAMTKKMC -52
HS_PAQR3      MHQKLLKSAHYIELGSYQYWPVLVPRGIRLYTYEQIPGSLKDNPYITDGYR-AYLPSRLC -60
              *::: **.       .:: :        ** : .::  :* :* *: : ** . :..::*

PAQR-3        AKSAFHINNETINIWSHLLGFIYFTYQQYNTNYIVLPSIGSHPSDHFVFTLSIFGMQMCM -112
HS_PAQR3      IKSLFILSNETVNIWSHLLGFFLFFTLGIYDMTSVLPSASASREDFVICSICLFCFQVCM -120
               ** * :.***:*********: *          **** .:  .*..: ::.:* :*:**

PAQR-3        LLSASYHTFGCT-SIEMRQKWLKMDIFGISAGLLGMYLNGIYTAFFCFQDHLTSYIYILL -171
HS_PAQR3      LCSVGYHLFSCHRSEKTCRRWMALDYAGISIGILGCYVSGVFYAFYCNNYWRQVYLITVL -180
              * *..** *.*  * :  ::*: :*  *** *:** *:.*:: **:* :     *:  :*

PAQR-3        GIFVITAYVPTRQDFFERKIIGSRVGLLHIIYCIIITFGICPTVHWVFLHGGFENDHVTK -231
HS_PAQR3      AMILAVFFAQIHPNYLTQQWQRLRS----IIFCSVSGYGVIPTLHWVWLNGGIGAPIVQD -236
              .::: . :.  : ::: ::    *     **:* :  :*: **:***:*:**:    * .

PAQR-3        WFPNVVVLYSLIAAAFMFYVTMVPEKLWPGKFDVVGCSHQWWHIFILGAMIYWQQSGNQL -291
HS_PAQR3      FAPRVIVMYMIALLAFLFYISKVPERYFPGQLNYLGSSHQIWHILAVVMLYWWHQS--TV -294
              : *.*:*:* :   **:**:: ***: :**::: :*.*** ***: :  : :*:**   :

PAQR-3        LTEYRSFTDSCHRFIPRNFSEISSSSVNSNYSNSPAM                        -328
HS_PAQR3      YVMQYRHSKPCPDYVSHL-------------------                        -312
               .    .:..*  ::.:
```

FIG S2

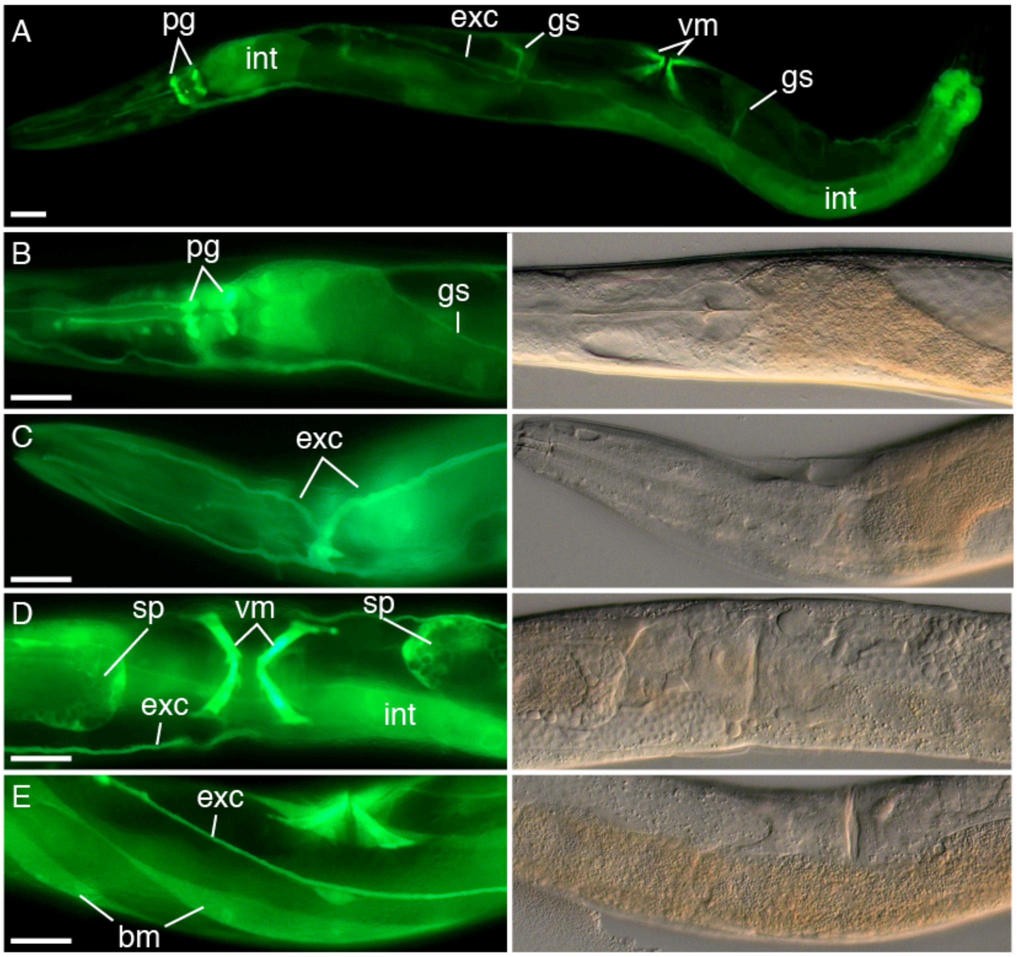

FIG S3

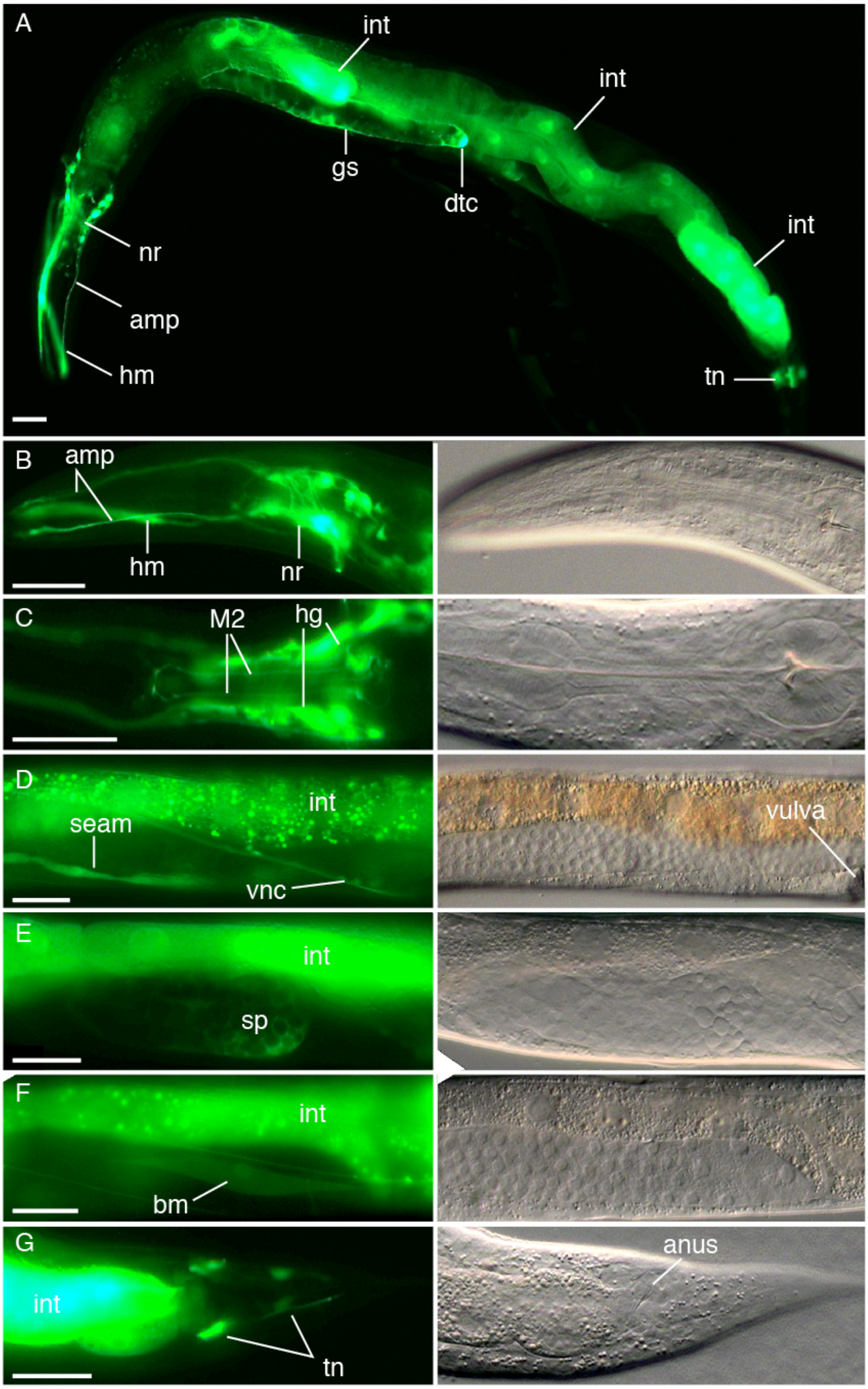
A
int
int
gs
dtc
int
nr
amp
hm
tn
B
amp
hm
nr
C
M2
hg
D
int
seam
vnc
vulva
E
int
sp
F
int
bm
G
int
tn
anus

FIG S4

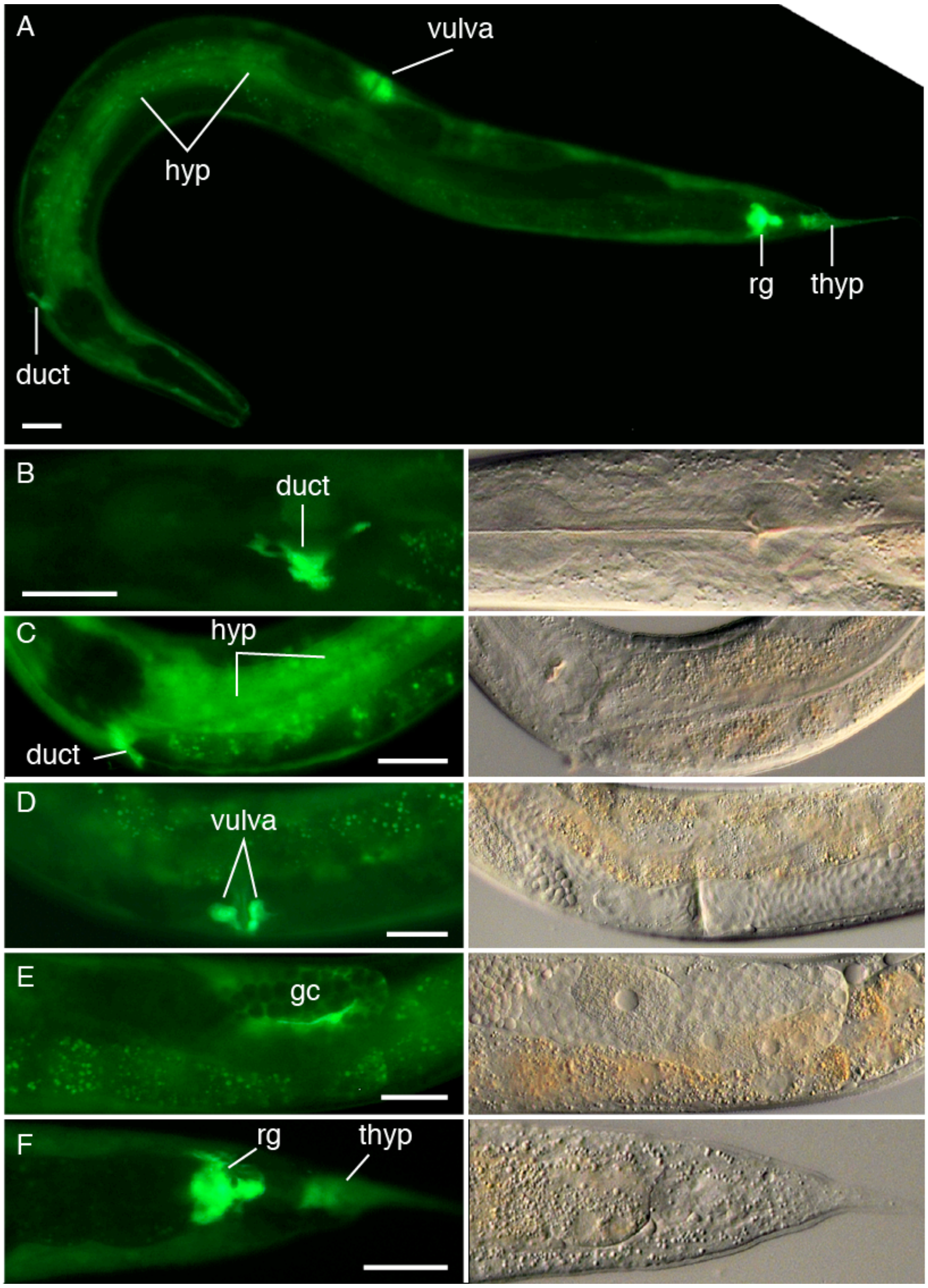

FIG S5

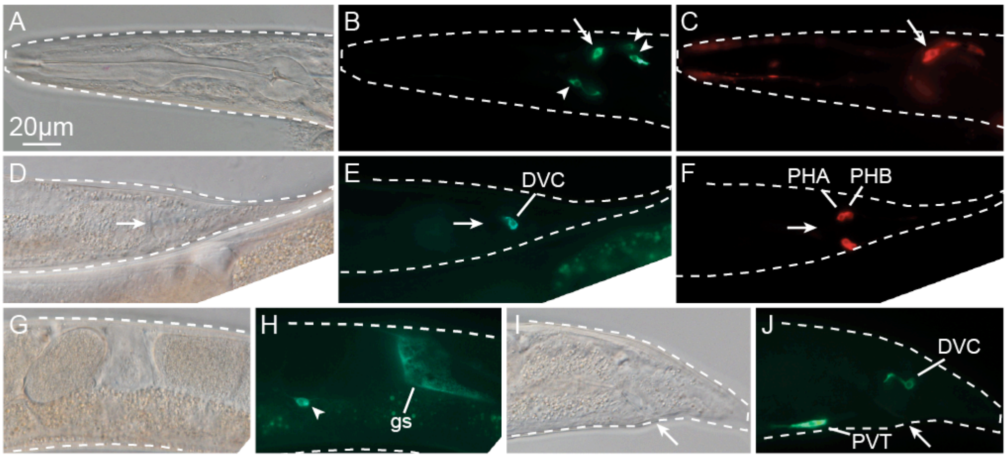

FIG S6

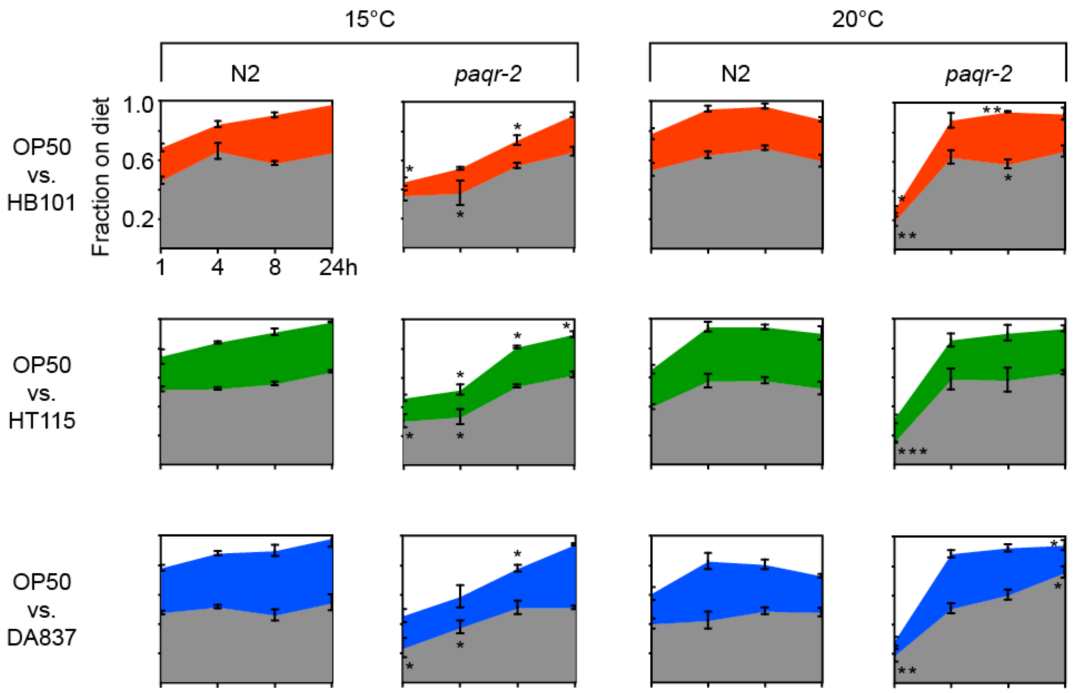
15°C
20°C
N2
paqr-2
N2
paqr-2
OP50
vs.
HB101
OP50
vs.
HT115
OP50
vs.
DA837
Fraction on diet
1.0
0.6
0.2
1
4
8
24h

Table S1: Double mutants tested for possible genetic interaction with *paqr-1* or *paqr-2*.

	**Gene name**	**Systematic gene name**	**Allele**	**Type of mutation and reference (unreferenced details are from WormBase)**	**Homolog**	**Result**
***paqr-1* background**						
Signaling:	*sbp-1*	Y47D3B.7	ep79	2181bp deletion of exons 7-10 and thus the C-terminal regulatory regions, reduction of function allele (4).	SREBP	Synthetic lethal. 16 out of 97 (16.5%) randomly picked progeny from paqr-1 homozygous;sbp-1 heterozygous hermaphrodites were detected as double mutants by PCR. None of these produced any progeny.
	nhr-49	K10C3.6	gk405	1275bp deletion, deletes 791bp of 5' UTR and all of exon 1.	PPARα (functional)	Viable
	aak-2	T01C8.1	ok524	408bp deletion, null (5).	AMPK	Viable
***paqr-2* background**						
Signaling:	*sbp-1*	Y47D3B.7	ep79	see above	SREBP	Synthetic lethal. 21 out of 102 (20.6%) randomly picked progeny from paqr-2 homozygous;sbp-1 heterozygous hermaphrodites were detected as double mutants by PCR. None of these produced any progeny.
	nhr-49	K10C3.6	gk405	see above	PPARα (functional)	Synthetic lethal. 18 out of 100 (18.0%) randomly picked progeny from paqr-2 homozygous;nhr-49 heterozygous hermaphrodites were detected as double mutants by PCR. Amongst the double mutants 4 out of 18 (22.2%) produced 1-2 progeny, however, non of these were able to found a line.
	nhr-80	H10E21.3	tm1011	446bp deletion, likely loss of function (6).	Worm specific (HNF4)	Rescue growth at 15°C
	aak-2	T01C8.1	ok524	see above	AMPK	Rescue growth at 15°C
FA synthesis:	*fat-5*	W06D12.3	tm420	779bp deletion, likely null (6).	Δ9 desaturase	Viable

	fat-6	VZK822L.1	tm331	1232bp deletion/ 428 bp insertion, likely null (6).	Δ9 desaturase	Synthetic sterile. 29 out of 99 (29.3%) randomly picked progeny from paqr-2 heterozygous;fat-6 homozygous hermaphrodites were detected as double mutants by PCR. All of these grew to adulthood but none produced any progeny.
	fat-7	F10D2.9	wa36	C to T substitution, minimum strong reduction of function (6).	Δ9 desaturase	Viable
β oxidation:	*acs-2*	F28F8.2	ok2457	1868bp deletion, eliminates exon 1-3 and part of exon 4.	Acyl-CoA synthetase	Viable
Ceramide synthesis:	*hyl-1*	C09G4.1	ok976	863bp deletion, loss of function (7).	Ceramide synthase	Viable
	hyl-2	K02G10.6	ok1766	1626bp deletion, eliminates exons 2 to 5 corresponding to 74% of the coding sequence (7).	Ceramide synthase	Viable
	lagr-1	Y6B3B.10	gk327	200bp deletion, loss of function (7).	Ceramide synthase	Viable
	F27E5.1	F27E5.1	ok564	868bp deletion/ 6bp insertion, deletes 417bp of 5'UTR, all of exon 1 and 2, 63% of exon 3.	Acid ceramidase	Viable
